# A theoretical systematic review of patient involvement in health and social care education

**DOI:** 10.1007/s10459-022-10137-3

**Published:** 2022-07-16

**Authors:** Amber Bennett-Weston, Simon Gay, Elizabeth S. Anderson

**Affiliations:** The George Davies Centre, Leicester Medical School, College of Life Sciences, University of Leicester, University Road, Leicester, LE1 7RH UK

**Keywords:** Theoretical quality, Patient involvement, Professional education, Systematic review

## Abstract

Patient involvement in health and social care education lacks theoretical underpinning, despite increasing calls for rigour. Theories help explain how learning is advanced and offer guidance for how faculty work with patients who become involved in curriculum delivery. We conducted a systematic review to synthesise how theory shapes our understanding of patient involvement in health and social care education. Three databases were systematically searched. Studies demonstrating explicit and high-quality application of theory to patient involvement in teaching and learning or involvement within a community of health and social care educators, were included. A narrative synthesis was undertaken using Activity Theory as an analytical lens to highlight the multifaceted components of patient involvement in professional education. Seven high-quality, theoretically underpinned studies were included. Four studies applied theory to pedagogy, showing how deep learning from patient involvement occurred. Despite a growing body of studies which attempt to use theory to explain learning, many were descriptive, lacked theoretical quality and were therefore excluded. Three studies applied theory to illuminate the complexity of involving patients in the educational system, showing how patients can be supported and valued in teaching roles. This review highlights that more work is required to identify the mechanisms through which patient involvement enhances learning and, to explore what involvement within the education community means for faculty and patients. Our understandings of patient-educator partnerships for learning could be progressed by further high-quality theory driven studies, which include the patient voice.

**Supplementary Information:**

The online version contains supplementary material available at 10.1007/s10459-022-10137-3.

## Introduction

Medical educators continue on a journey to advance and understand how to frame the involvement of patients within programme delivery systems (Regan de Bere & Nunn, 2016). In this review, the term “patient(s)” will be used to describe those who represent their own lived experience of health and social care, and carers. We recognise that this term has been associated with paternalistic power relations between professionals and patients. However, alternatives such as service user or client, are also contested. While it remains common sense that medical education involves patients, early thinking mirrored the paternalistic paradigm of medical knowledge control and resulted in using the public as a learning resource (Malterud et al., 2004; Spencer et al., 2011). Today this position has largely been eroded as we work together towards an equality and equity agenda where patients are involved in curriculum co-creation, teaching, student selection and assessment (Spencer et al., 2011; Towle et al., 2010). This reflects modern patient-centred team working and aspirations for integrated care where patients are more involved in their health and social care journey and the system design (Department of Health, 2000, 2001, 2005, 2007, 2009; Department of Health & Social Care, 2021; Health Council of Canada, 2006; Institute of Medicine, 2000). These changes are steeped in the social movement of disability and reports of quality and safety failings (Beresford & Croft, 2016; Francis, 2013; Oliver, 2013).

Consequently, medical education has advanced rapidly to ensure this partnership agenda informs the foundations of student learning (Towle et al., 2010), propelled by professional bodies such as the General Medical Council (GMC) and modern policy (Department of Health & Social Care, 2021; GMC, 2009; 2018). Involvement is now conceptualised as a spectrum, ranging from minimal involvement in learning material development and storytelling roles to equal partnerships between patients and educators (Towle et al., 2010). As envisaged in the early citizen participation movement (Arnstein, 1969), partnership should be the ultimate goal of involvement initiatives, to benefit the entire community and add credence to patients’ authenticity (Gordon et al., 2020).

At this juncture, several reviews have crystallised the many roles for patients in medical education and the undoubtedly transformational effects which support their involvement (Dijk et al., 2020; Gordon et al., 2020; Spencer et al., 2011; Towle et al., 2010). Recent reviews show that despite an increase in activity, and amidst critical discussions on the positioning of patients within medical education (Sebok-Syer et al., 2021), most initiatives involve patients as storytellers and there is no evidence of partnership relationships (Dijk et al., 2020; Gordon et al., 2020). While in other areas of professional education – notably social care, nursing and mental health – storytelling without partnership relationships continues (Morgan & Jones, 2009; Robinson & Webber, 2013; Terry, 2012), there are examples which extend our understanding of new possibilities beyond storytelling roles, including exploring patient involvement in leading teaching roles, curriculum design, student selection, and assessment (Anderson et al., 2019; Downe et al., 2007; Happell & Roper, 2009; Rhodes & Nyawata, 2011; Simons et al., 2007; Unwin & Rooney, 2020).

To enhance faculty development, educators must understand how such advancing pieces of work can help us to conceptualise the involvement of patients and carers in professional education, going beyond acknowledgement of the many challenges, both practical and systemic, for appropriate infrastructure, resources, and faculty support (Basset et al., 2006). Regan de Bere and Nunn (2016) proposed Activity Theory (Engeström, 2001) as an analytical heuristic to explore the involvement of patients in medical education. Activity Theory has roots in psychological and cultural-historical traditions and conceptualises human activity as a system comprising various factors that interact to achieve specific outcomes (Engeström, 2001). Activity Theory can capture the complexity of integrating patients within programme delivery systems; nevertheless, there remains a need for theoretically grounded, empirical understandings of the ‘multiple roles and realities’ of patient involvement in professional education (Regan de Bere & Nunn, 2016).

Literature reviews can propel our understanding and recent reviews have highlighted that published descriptions and evaluations of patient involvement in medical education lack theoretical underpinning (De Groot et al., 2020; Dijk et al., 2020; Gordon et al., 2020). Theories generate knowledge that can be generalised across settings and are the foundation of conceptual frameworks which illuminate and magnify explanations for how and why complex activities work (Bordage, 2009; Reeves & Hean, 2013; Sutton & Staw, 1995). In the context of patient involvement in medical education, theory can enhance our understandings in two ways; first, theoretically underpinned teaching and learning illuminates curricular assumptions, justifies curricular practices and provides a basis for future iterations and testing (Bordage, 2009; Kamel-El Sayed & Loftus, 2018) and second, theory can illuminate what it means to work in partnership with patients within the education community (Gordon et al., 2020; Regan de Bere & Nunn, 2016). Without theoretical roots we are unlikely to advance (Gordon et al., 2020).

Recent reviews (De Groot et al., 2020; Dijk et al., 2020; Gordon et al., 2020) have not looked beyond medical education, possibly missing learning from other professions and interprofessional education (IPE). More importantly, these reviews have not taken a systematic approach to searching for and synthesising theory, nor have they used a dedicated theoretical quality appraisal tool to assess the quality with which theory has been applied in each study. Theoretical reviews, which seek to explore the effective use of theory, address these issues and are an important resource to support health and social care educators (Hean et al., 2018). The use of a tool to assess theoretical quality (Hean et al., 2016) ensures theory has been applied and aligned throughout a study to reveal deeper understandings. There has not yet been a theoretical review of the literature on patient involvement in any area of professional health and social care education, despite increasing calls for theoretical perspectives (Regan de Bere & Nunn, 2016).

The purpose of this theoretical review is to synthesise the contribution that high-quality theoretical papers have made to patient involvement in professional education, to illuminate best practice. As patient involvement is well-established in disciplines like social care, nursing, and mental health (Spencer et al., 2011), we include studies from all areas of health and social care education. This is the first review of theory to be conducted in this field.

We asked the following questions:

- How is theory framed and applied to patient involvement in teaching and learning in professional health and social care education?
- How is theory framed and applied to the integration of patients into an educational community and their involvement in the faculty?

## Method

### Design

Following the fact that there is a well-established body of literature in this field, the subject of this study goes beyond a scoping review, which is typically used to explore emerging evidence (Munn et al., 2018), and requires a systematic review. We conducted a systematic review in accordance with the guidelines of the Associations for Medical Education in Europe (AMEE; Sharma et al., 2015). The reporting of the review is guided by the STORIES (STructured apprOach to the Reporting In healthcare education of Evidence Synthesis) statement (Gordon & Gibbs, 2014). The review was registered in PROSPERO (CRD42021230682).

### Search strategy

We searched the electronic databases MEDLINE, CINAHL and PsychINFO for papers in English between January 2000 and December 2020. This timeframe correlates with the introduction of a partnership agenda in health and social care with the ‘New NHS’ (Department of Health, 2000). Search terms were guided by recommendations for systematic searching for theory, adapting Booth and Carroll’s (2015) BeHEMoTh framework (Behaviour; Health condition or context; Models or Theories; Online Appendix 1) and previous systematic reviews (Dijk et al., 2020; Gordon et al., 2020). Authors of unavailable papers were contacted; those that did not respond were excluded. Hand searching and ancestry searching, which involves searching the reference lists of relevant papers (Haig & Dozier, 2003), were conducted to identify further papers.

### Eligibility criteria

Our criteria sought papers showing an active patient involvement in professional education where theory was explicitly referenced and applied (Table 1). In alignment with Gordon et al. (2020), we only included studies that discussed patient involvement in more than a cursory fashion, providing detail beyond a single statement about patient involvement. This was important given our aim to identify best practices for patient involvement. Involvement followed Towle et al. (2010) Spectrum of Involvement which considers active involvement as beyond incidental patient-student encounters to planned participation in teaching. We excluded papers where patients were acting as others and not themselves. Our definition of ‘theory’ aligns with Hean et al. (2016): excluding non-conceptual frameworks and models which state what was done rather than how and why. After discussion we included papers using participatory-action-research (PAR), because of evidence that PAR generates and tests action theories (Greenwood, 1994). To be eligible for inclusion, papers applying PAR had to explicitly articulate its theoretical foundations.

**Table 1:** Eligibility criteria

Inclusion criteria	Exclusion criteria
Papers that report on patient involvement in professional health and social care undergraduate education, postgraduate education, or continuing professional development	Papers that report on patient involvement outside of an educational context (i.e., research or practice)
Papers that report on *active* patient involvement in planned, formal, curriculum embedded educational activities, or any other planned faculty work (i.e., Any involvement which can be categorised using the Spectrum of Involvement) **OR**	Papers that explore incidental learning encounters between health and social care students and patients without reference to planned involvement in faculty work (i.e., Any involvement that cannot be categorised using the Spectrum of Involvement) **OR**
Papers that report on the preparation, integration, or support of those patients who are *actively* involved within an educational community	Papers that do not report on the preparation**,** integration**,** or support of those patients who are *actively* involved within an educational community
Papers that provide comprehensive detail on the nature of the patient involvement taking place	Papers that discuss patient involvement in a cursory fashion, with no detail given to judge the nature of involvement taking place
Papers where ‘patients’ involved in education represent their true selves and lived experience	Papers where ‘patients’ are people who are trained to portray a simulated scenario/case and do not represent their true selves or lived experience (i.e., simulated patients)
Papers that reference theory as underpinning the design, development, or evaluation of patient involvement in educational activities and/or the preparation, integration, or support of patients within an educational community	Papers which discuss non-theoretical models or frameworks without explicit alignment to a specific theory
Papers published between January 2000 and December 2020	Papers published earlier than 2000 or after December 2020
Papers published in English	Papers which are not published in English
Full-text primary empirical research and non-empirical research	Grey literature, theses, policy documents and book chapters, or empirical and non-empirical research where the full-text article is not available and contacted authors do not respond

### Initial selection

After retrieving the search results from the different databases, duplicates were removed using RefWorks, a web-based bibliography and database manager. Once duplicates were removed, the titles and abstracts of the resulting citations were screened by all three reviewers (ABW, SG, EA). At this stage, we retained all articles that explicitly referenced the application of theory to patient involvement in professional education and those that referenced involvement in education alone, to ensure capture of all potentially eligible studies. The second stage of screening involved checking the full texts of all retained studies against the eligibility criteria, along with papers retrieved through hand and ancestry searching. To ensure reliability we discussed a sample of papers (n = 10) as a team. Those papers that met the eligibility criteria were taken forward for the final stage of selection and for further assessment of theoretical quality.

### Assessment of theoretical quality

In a conventional review of the evidence-base, the assessment of methodological quality is a core process in selecting sufficiently rigorous studies to constitute good evidence (Hean et al., 2016, 2018). Where the review focus is theory, this should be reframed to assess the quality with which theory has been applied; only papers that have applied theory with the greatest quality, should be included in a theoretical review (Hean et al., 2016). We adapted a Theoretical Quality Tool (Online Appendix 2), initially developed by Hean et al. (2016) for application within a Best Evidence in Medical Education (BEME) review of the contributions of theory to IPE (Hean et al., 2018). The tool assesses five dimensions of theoretical quality based upon Fawcett and Downs’ (1992) work on the link between theory and research: pragmatic adequacy, does the theory have clear utility for practice; parsimony and testability, is the theory clearly described and propositions derived from that theory clearly presented; and operational and empirical adequacy, considering whether there are clear links between the theory, the research methods used, and the empirical data collected (Hean et al., 2016).

The tool has a maximum score of 9, with three items assessing pragmatic adequacy, three items assessing parsimony and testability, and for empirical papers only, three items assessing operational and empirical adequacy. To be eligible for inclusion in the final sample, papers had to demonstrate pragmatic adequacy and clear articulation of the theory they claimed to deploy (parsimony and testability), receiving a minimum score of 6/9 points on the theoretical quality tool (Hean et al., 2018) from each of the three authors (ABW, SG, EA). Papers that included sophisticated theoretical discussions but lacked application to actual curricular practices or patient involvement, were considered ‘aspirational’ and were excluded. All authors (ABW, SG, EA) initially worked together on five papers until a shared understanding of high-quality theory had been reached, before independently applying the tool to each of the remaining retained papers. We met regularly as a team to share and exchange our progress. Complete agreement between all authors (ABW, SG, EA) on inclusion/exclusion was achieved for the majority of papers. For a small subset of papers, we sought clarification regarding inclusion/exclusion through discussion.

### Data extraction

A data extraction template was constructed in Microsoft Word^®^ (2018) to cross-reference the nature of the patient involvement and/or educational intervention reported, with the application of theory. This was piloted on one paper by each reviewer and discussed as a team to ensure completeness.

Initially, we had intended to capture the ‘level’ of involvement reported in each included paper, using the well-established ‘Spectrum of Involvement’ (Towle et al., 2010). However, similar to Gordon et al. (2020) we encountered difficulties; none of the involvement initiatives reported fitted neatly, or even roughly, into one specific level. The same problem presented itself upon turning to the equally well-established ‘Ladder of Involvement’ (Tew et al., 2004). As such, we were unable to definitively align each paper to one prescriptive level. These challenges may indicate issues with the utility and applicability of such tools in ‘real-life’ educational practice, though it is also possible that educators are simply not taking these tools into account when planning or reporting their involvement initiatives.

### Data synthesis

**Fig. 1:**
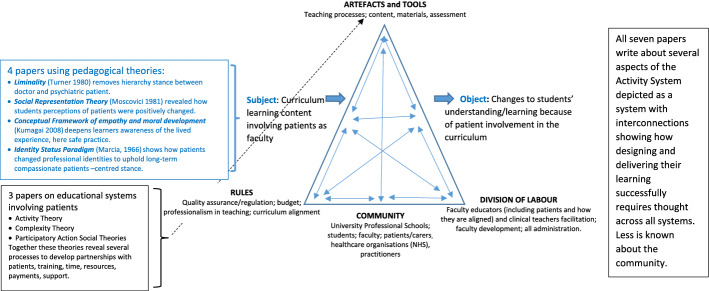
Theories used in patient involvement applied to activity theory

We conducted a narrative synthesis on the final sample of included studies (Popay et al., 2006). Activity Theory (Engeström, 2001) was applied as an analytical framework to consider the multifaceted components within training schools that must come together to deliver teaching to health professions students, the *Subject*, for learning, the *Object,* to achieve the broader *Outcome* of meeting curriculum requirements for registration as a practitioner*.* In the Activity System for patient involvement in professional education (Fig. 1), the *Subject* and *Object* are enhanced because of the involvement of patients (Regan de Bere & Nunn, 2016). The final sample was analysed to consider the theoretical contributions made by these high-quality, theoretical papers to the components of Activity Theory, namely: the mediating *Artefacts and Tools* (all the support processes), the *Rules* (regulator policies, standards, and ethical codes), the education *Community* (employed and peripheral members, and their sense of shared vision) and *Division of Labour* (how teaching is managed and orchestrated). Activity Theory reflects the richness and multifaceted nature of the complex system of any Higher Education Institution (HEI) and is a suitable framework against which we can begin to see the gaps in our theoretical understanding of patient involvement in professional education (Regan de Bere & Nunn, 2016).

## Results

**Fig. 2:**
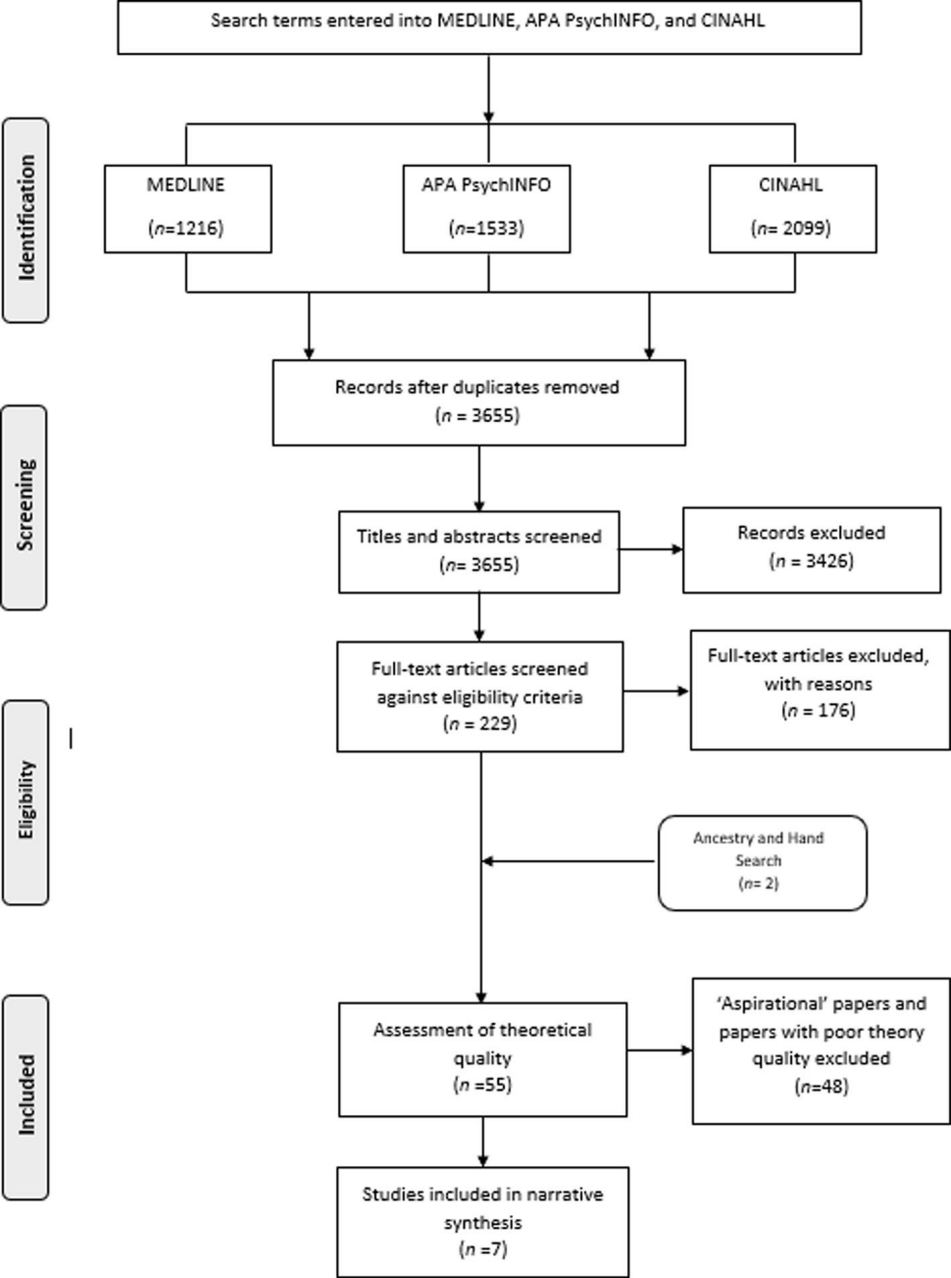
Study flow diagram

The final searches produced a total of 4848 citations. After removing duplicates, the resulting 3655 citations were available for title and abstract screening. 229 papers were retained and checked, in full, against the eligibility criteria, followed by an additional 2 papers retrieved through hand and ancestry searching. 55 papers met the eligibility criteria and were taken forward for the assessment of theoretical quality by all three authors. Only papers demonstrating theoretical sophistication were included. The degree of agreement on inclusion/exclusion between all authors (ABW, SG, EA) at this stage was approximately 91%. Conflicts between reviewers’ judgments on the remaining 9% of the papers assessed for theoretical quality were resolved through discussion. This left a final sample of 7 high-quality theoretically underpinned papers, all of which scored the maximum 9/9 points on the Theoretical Quality Tool (Hean et al., 2016). Figure 2 provides an overview of how the final sample was reached.

### Overview of included studies

The final 7 high-quality theoretical papers were published between 2006 and 2020, most (n = 5) within the last two years and were from Europe and Canada (Table 2). Four papers focused on uni-professional student groups: two were undergraduate in medicine (Kline et al., 2020) and pharmacy (Hache et al., 2020), and two were postgraduate in medicine (Jha et al., 2015) and psychiatry (Agrawal et al., 2020). Two studies were interprofessional including medicine, speech and language therapy, midwifery, nursing, physiotherapy, occupational therapy, and social work (Anderson et al., 2019; Cooper & Spencer-Dawe, 2006). One paper focused on patients involved across a faculty of medicine and health sciences (Read et al., 2020). All but one paper (Hache et al., 2020) stated whether the teaching was mandatory (Agrawal et al., 2020; Anderson et al., 2019; Jha et al., 2015) or elective (Cooper & Spencer-Dawe, 2006; Kline et al., 2020). Patient involvement occurred in the classroom (Anderson et al., 2019; Cooper & Spencer-Dawe, 2006; Hache et al., 2020; Jha et al., 2015); with two in community settings (Agrawal et al., 2020; Kline et al., 2020). Four papers adopted a qualitative methodology (Agrawal et al., 2020; Anderson et al., 2019; Cooper & Spencer-Dawe, 2006; Kline et al., 2020), two papers a mixed-methods approach (Hache et al., 2020; Read et al., 2020), and one reported a randomised controlled trial (RCT; Jha et al., 2015). Although we were unable to definitively assign a level of involvement, most studies demonstrated concerted efforts to work towards this through consideration of patient payment, training, support, and involvement beyond curriculum delivery.

**Table 2:** Description of final sample*

Author(s) and year	Country	Study design	Professional group(s)	Learning environment	Mandatory or elective?	Intervention/patient involvement	Support for patient involvement	Theory: what and why?
Agrawal et al. (2020)	Canada	Qualitative	Postgraduate Medicine: Psychiatry	Various community settings	Mandatory	Patient ‘advisors’ partner with residents meeting monthly over 6 months to share experiences of mental illness	Small honorarium provided for patients’ participation Monthly supervision meetings held for patients by course directors	*Liminality* To understand participants’ experiences and explain how a co-produced pedagogy mechanistically achieves its effects on student learning
Anderson et al. (2019)	UK	Qualitative	Undergraduate Midwifery; Speech and Language Therapy; Nursing; Medicine; Social Work	Academic	Mandatory	Explored perceptions of patients, students, and teachers on progressing involvement from storytelling to leading teaching roles in interprofessional education	Training and support for patients involved in teaching Remuneration system in place	*Activity Theory* To explore what it means for all stakeholders to progress patients into leading teaching roles
Cooper and Spencer-Dawe (2006)	UK	Qualitative	Undergraduate Medicine; Nursing; Physiotherapy; Occupational Therapy; Social Work	Academic	Elective (All students encouraged to attend)	Patients co-facilitate 2–4 interprofessional education workshops, sharing their lived experiences	Training for involvement Support from independent organisation during involvement Expenses for training and payment for involvement provided	*Complexity Theory* To underpin development of the intervention and analyse patient involvement in interprofessional education
Hache et al. (2020)	France	Mixed-Methods	Undergraduate Pharmacy	Academic	Not specified	Patients involved in the development and delivery of a 2-h workshop on patient education programmes. Patient ‘partner’ facilitated workshop discussions without faculty mediation and provided two essential learning points at the end of the workshop	No training, support, or payment for patients reported	*Social Representations* To explain students’ learning on patients’ expectations of the pharmacist’s role in patient education programmes
Jha et al. (2015)	UK	RCT	Postgraduate Medicine	Academic	Mandatory	Patients collaborate in developing the intervention which consists of two 1 h long sessions where they share stories of inadequate care or medical error. A facilitated discussion between patients and students follows	Preparation for involvement Emotional support and de-briefing provided post involvement No payment reported	*Kumagai’s Conceptual Framework of Empathy and Moral Development* To underpin intervention and explain students’ learning on patient safety
Kline et al. (2020)	Canada	Qualitative	Undergraduate Medicine within interprofessional curriculum	Various community settings	Elective	Patient ‘mentors’ partner with small student groups 2–3 times over 16 months to teach on their experiences of chronic illness or disability	Support meeting midway through module No training or payment reported	*Identity Status Paradigm* To explain how learning with patients acts to shape students’ professional identity
Read et al. (2020)	UK	Mixed-Methods	Health Faculty	N/A	N/A	Patients involved as partners in co-producing, delivering, and evaluating an educational development programme for patients involved in education across a Health Faculty	Development, delivery, and evaluation of training programme for patients involved in education, with patients	*Participatory Action Research* To provide a framework for co-producing an educational programme for patients involved in education

*Theory denoted by italicised text

In alignment with our research questions, the 7 papers fell into two broad categories based on their application of theory. Four papers used theory to explain the mechanism through which patient involvement enhances students’ learning (Agrawal et al., 2020; Hache et al., 2020; Jha et al., 2015; Kline et al., 2020), while three papers applied theory to explain patient alignment within an educational community (Anderson et al., 2019; Cooper & Spencer-Dawe, 2006; Read et al., 2020). The synthesis presented below explains how the theories used in these papers contribute to the different aspects of the Activity System for patient involvement in professional education.

### Components of the activity system where theory contributes

The seven high-quality, theoretical papers were aligned to the components involved in the Activity System for patient involvement in professional education. We can begin to see the aspects of the system to which high-quality theory has been applied, and those which require greater theoretical consideration (Table 3). The four papers that focus on the teaching and learning process illuminate the *Subject-Object* pathway (Agrawal et al., 2020; Hache et al., 2020; Jha et al., 2015; Kline et al., 2020), and the three papers that explore the integration of patients into the educational community highlight the complexity issues within the triangle of *Rules, Artefacts and Tools, Community* and *Division of Labour* (Anderson et al., 2019; Cooper & Spencer-Dawe, 2006; Read et al., 2020).

**Table 3:** Synthesis of final sample to Activity Theory*

Papers	Subject	Object	Community	Artefacts and tools	Rules	Division of labour
1. Agarwal et al. (2020)	Fourth year psychiatry residents working alongside a patient advisor to explore mental illness and recovery. The learning objectives were co-produced	Transformation of the learner through the process of the theory of *Liminality*. The learning objectives were met using a constructivist transformative and reflective pedagogy	The work led to a shift in power between psychiatrist/residents and advisor/patient. The course was relationship building	Monthly supervision meetings for the advisor patients in mutually agreed places for safe non-judgemental environments		Longitudinal alignment of a learning partnership meeting monthly over a six-month period. Developed a relationship of trust. Changed relationships by shifting power dynamics
2. Anderson et al. (2019)	Patients become teachers in IPE workshop looking at what this means for all stakeholders across the whole system of learning using *Activity Theory*		While many patients wish to progress from telling their stories some patients do not wish to take on leading teaching roles and these thoughts were reflected by students. Need to build trust for all stakeholders where patients lead teaching	Support processes for patients in leading teaching roles—resources to offer training for patients. Patients required training and support for leading the class/small group; training concerning policy, professionalism in teaching, diversity being part of steering groups	Patients to become co-tutors working with an experienced teacher/practitioner Patients paid; remuneration system put in place Patients who lead teaching must be trained in order to build trust for all stakeholders	Patients can work as Co-Teachers. Mentor roles were needed to support new patients and those wishing to go on to become leaders as co-tutors in this workshop. Faculty needed to be supporters to co-tutors in the teaching
3. Cooper and Spencer-Dawe (2006)	IPE event involving trained patient teachers. Students’, patients’, and educators’ experiences of IPE event analysed through lens of *Complexity Theory*	Patient involvement advanced students’ understandings of patient-centred care and helped them make connections between theory and practice	The workshop established interprofessional team working between patients, carers, and educators However, building a learning community is complex and requires multiple components to be considered to ensure all stakeholders feel confident and safe, and benefit	Certain tools identified to support patient involvement in IPE Despite joint training on IPE facilitation, educators need specific training on working with patients in education as opposed to clinical practice Patients need training on IPE co-facilitation and aims, and support in administrative tasks and HEI building navigation	Patients valued the direct and open payment approach Students need patients’ roles to be well defined and feel patients should be trained to uphold teaching quality Patients need greater role clarity as ‘co-facilitator’ Educators feel that a clear person specification is needed to support patient recruitment and strengthen both partnerships and IPE quality	Patient positioned as co-facilitators to educators and equal members of interprofessional team External patient-led organisation recruited patients for involvement and supports during
4. Hache et al. (2020)	Undergraduate Pharmacy students learning from patient partners about patient education programmes	Students’ understandings on the role of the Pharmacist in patient education programmes advanced post-intervention and explained through the theory of *Social Representations*			Confidentiality addressed at the start of the workshop to protect patients involved	Workshop on Patient Education programmes developed by educators alongside patients Patients as co-facilitators of workshop with educators
5. Jha et al. (2015)	Patients’ narratives deployed in a patient safety intervention for Foundation Year 1 Medical Trainees	Trainees’ depth of understanding on patient safety enhanced through patient narratives and explained using *Kumagai’s Conceptual Framework of Empathy and Moral Development,* as a process of transformation			Preparation workshops for patients to create a supportive confidential environment in which to identify which aspects of their narratives were important for trainees’ learning Emotional support and debriefing provided for patients given distressing nature of their stories	Patients lead learning; following discussion facilitated by researchers/educators. Support provided by researcher
6. Kline et al. (2020)	Patient mentors teach medical students about their experiences of health and social care as part of IPE curriculum	Medical residents form patient-centred professional identities 3–4 years after IPE programme. This is attributed to patient involvement in preclinical IPE and explained by concepts from *Identity Status Paradigm*	The course fostered longitudinal relationships between patient mentors and medical students in a safe environment	Optional mentor support midway through programme	Pre-programme orientation for all mentors and students Patient recruitment policies developed by steering committee involving patients	Longitudinal learning partnership in preclinical IPE over 16 months Patient as mentor, medical student as learner. Educators absent at meetings but set discussion topics
7. Read et al. (2020)			Faculty-wide training developed with patient involvement viewed by patients as expression of their value to the University and combats feelings of isolation within each respective school	University investment of resources required to support patients involved in education to ensure ultimate engagement and continuity and affirm value Educators need to invest time to facilitate new patient training	Patients involved in the development, delivery, and evaluation of a training programme through *Participatory Action Research* Unmet training needs identified and incorporated into programme e.g., IT, communication, and interview skills, participation in meetings, and introduction to HEI	Patients involved as partners in all aspects of the training Academic staff act as co-facilitators in this Scope for patients to progress and take on bigger roles in training delivery to reduce time-burden on academic staff

*Theory is denoted by italicised text

### Subject-object pathway

Agrawal et al. (2020) interpreted their analysis of a co-produced pedagogy for fourth year psychiatric residents through the theoretical lens of *liminality* (Turner, 1980). The authors offered the five intersecting and mutually reinforcing concepts of *liminality* (the *betwixt and between* space, *neophyte* and *elder,* communication of *sacra*, *liminal reflexivity*, *normative order of life*, and *transformation*) to explain how patient involvement transformed learners’ understandings of, and clinical approaches to, mental illness recovery. These concepts show how engaging patients as ‘advisors’ to psychiatric residents outside of traditional clinical learning settings can disrupt the embedded, dominant power relations between psychiatrist-to-be and patients. This sets in motion a process of knowledge sharing and reflection and ultimately brings about transformative learning. The theory of *liminality* emphasises and explains the value of challenging the implicit hierarchies embedded in psychiatric training by positioning patients as ‘advisors’ for residents’ learning.

Hache et al. (2020) applied *social representations theory* (Moscovici, 1981) to illustrate how patient partners can enhance undergraduate pharmacy students’ learning. Patient facilitated discussions provided essential learning points resulting in different representations of knowing post-course to pre-course. By showing that students’ *social representations* changed after being taught by patients, the authors demonstrate the profound impact that patients can have on students’ learning outcomes.

Using *Kumagai’s conceptual framework of empathy and moral development* (Kumagai, 2008) Jha et al. (2015) explain how patients’ stories of medical error worked to enhance Foundation Year 1 medical trainees’ learning on patient safety. In the UK, Foundation Year 1 is mandatory upon completion of an undergraduate medical degree to register with the GMC and practise as a doctor; this involves training in a series of rotations in different specialities within hospitals or the community. Adopting Kumagai’s framework, the authors deliberately deployed patients’ narratives. These narratives ‘communicated meaning’ by evoking both positive and negative emotional responses among the trainees, which the authors attribute to learners’ advanced understandings of the importance of communication in improving patient safety and increased positive attitudes towards patient involvement in reducing medical error. Both professional and patient teachers achieved the same outcomes but, the authors’ use of *Kumagai’s conceptual framework* demonstrates the importance of patients’ narratives and explains how they act to enhance learners’ depth of understanding of the meaning of medicine and the emotional response to medical error; ultimately allowing better identification with the patient’s perspective.

Only one study investigated the enduring impacts of patient involvement on learners. Kline et al. (2020) used Marcia’s (1966) *identity status paradigm* as a conceptual framework for the analysis of junior doctors’ experiences of an interprofessional ‘Health Mentor Programme’. The authors were interested in the long-term implications for learners’ professional identity of prior teaching from patient ‘mentors’. Findings revealed residents’ *commitments* to patient partnership, interprofessional collaboration and holistic care. These were rooted in the *exploration* of professional values and behaviours prompted by mentors’ storytelling and the development of longitudinal patient-student relationships during the programme. Through the *identity status paradigm,* Kline et al. (2020) reinforce the value of early exposure to the authentic and autonomous patient voice and illuminate how this can foster the development of long-standing patient-centred professional identities.

### The triangle of rules, artefacts and tools, community, and division of labour

Anderson et al. (2019) explored what it means for patients, carers and educators when patients progress into leading teaching roles in an established interprofessional workshop, through the lens of Engeström’s (2001) *Activity Theory.* All stakeholders supported a new role of patient ‘Co-tutor’ but identified factors necessary to build trust when patients lead teaching. These included patient training to develop competence and confidence in educational activities, student preparation to accept patients in authoritative positions, implementation of a patient remuneration system and faculty support for Co-tutors in teaching. A ‘Mentor’ role was highlighted as important to support new patients and those wishing to progress to Co-tutor. Through *Activity Theory,* Anderson et al. (2019) illuminated the complexity of moving patients beyond storytelling roles in ways that respects the needs of all stakeholders—a complexity compounded by the challenges of IPE.

Similarly, Cooper and Spencer-Dawe (2006) used *Complexity Theory* (Tosey, 2002) in their analysis of stakeholder data following an interprofessional workshop—also theoretically underpinned—involving trained patients as co-facilitators. They explain how outcomes developed through a process of *connectivity, self-organisation,* and *emergence*; patient involvement advanced students’ understandings of patient-centred care, bridging the theory–practice gap. Patients and educators also benefitted, reflecting the *a-linearity* of IPE. Working as an interprofessional team raised concerns for all, highlighting IPE’s *unpredictability*. Educators requested training on partnering with patients in education and patient recruitment specifications. Patients felt a lack of role clarity and required training on IPE objectives. Students worried about patients’ representativeness and teaching quality, reinforcing educators’ and patients’ concerns. Mirroring Anderson et al. (2019) findings, Cooper and Spencer-Dawe’s (2006) use of *Complexity Theory* illuminates that while beneficial, involving patients as teachers, particularly in IPE, is challenging.

Read et al. (2020) employed *PAR* to guide the development, delivery, and evaluation of a training programme for patients involved in educational activities across a faculty of medicine and health sciences. The *PAR* process highlighted numerous patient training needs unmet by school-specific programmes; these were addressed in the ensuing faculty-wide training programme which enhanced patients’ understandings of roles within the wider University, having implications for expanding recruitment and involvement. Patients viewed the training as an expression of value. Preparing and delivering training was labour intensive and time-consuming for educators. Ultimately, through *PAR*, Read et al. (2020) highlight the significance of working with patients on cross-faculty training to support and affirm the value of their involvement and ensure continuity. This is contingent on the investment of university resources and educators’ time and support.

## Discussion

Recent reviews on patient involvement in medical education show promising growth in the evidence-base but emphasise that the field is largely atheoretical (De Groot et al., 2020; Dijk et al., 2020; Gordon et al., 2020). This review set out to systematically search for and theoretically synthesise the theories applied to patient involvement in all areas of health and social care education, to identify how theory is helping to guide best practice. Seven high-quality, theoretically underpinned papers were identified. The included papers span undergraduate-to-postgraduate education and uni-professional to interprofessional teaching. Most studies were of undergraduate education, perhaps symptomatic of the recognition of the importance of shaping values and attitudes early in training (GMC, 2015; Gordon et al., 2020). Interestingly, the professions where patient involvement has been longer standing, social work and nursing, did not offer high-quality theoretical contributions. The majority of included papers were published within the last two years, reflecting a positive movement away from show and tell papers towards theorised questioning about how and why patients positively enhance student learning (Reeves & Hean, 2013). Only one paper (Kline et al., 2020) explored the longitudinal effects of involvement, supporting previous reviews highlighting this research gap (Dijk et al., 2020; Gordon et al., 2020; Spencer et al., 2011).

All of the included papers were awarded the maximum possible score on the Theoretical Quality Tool (Hean et al., 2016). This may be an indication that dimensions of theoretical quality are strongly interdependent; the papers with the most pragmatic adequacy tend to be those that clearly articulate the theory (parsimony), lay out clear propositions (testability) which are tested using appropriate methods (operational adequacy), and report data that is conceptually coherent with the theory that has been applied (empirical adequacy; Hean et al., 2016).

We applied Activity Theory as an analytical lens through which to synthesise the included papers, illuminating both the components of the HEI system where high-quality theory has been applied and those which need greater theoretical focus. As Engeström (2001) expected, this conceptual framework offers expansive learning concerning the current state of patient involvement as educational partners within modern health/social care programmes. Theory is predominantly applied to explain how patient involvement enhances learning; this tells us about the *Subject-Object* pathway (Agrawal et al., 2020; Hache et al., 2020; Jha et al., 2015; Kline et al., 2020). The use of theories of liminality (Turner, 1980) by Agrawal et al. (2020), transformative learning (Kumagai, 2008) by Jha et al. (2015), social representations (Moscovici, 1981) by Hache et al. (2020) and identity (Marcia, 1966) by Kline et al. (2020) explain how positioning patients as authoritative experts enhances learners’ understanding of the patient perspective. Although we have known for some time that patient involvement advances learning (Dijk et al., 2020; Spencer et al., 2011), these papers further contribute to our understanding by providing explanations for *how* and *why* an intervention was deployed and worked to achieve its effects.

We did identify numerous papers representing a vast range of learning theories (Online Appendix 3). However, these studies failed to align theory from the research aims to study design and evaluation; several simply referred to a theory by name, with no explanation of what the theory entails or how it was applied. These papers did not stand the test of our theoretical quality tool (Hean et al., 2016), scoring low/no points for clear articulation of theory (parsimony and testability) and operational/empirical adequacy, and were excluded. Although these papers confirm the depth of learning that can be achieved through involvement and thus fuel the agenda, further high-quality theoretically underpinned research is required if we are to begin to form an evidential agreement on best practices for developing, delivering, and evaluating education involving patients.

Our analysis using Activity Theory revealed that we know less about the HEI system as a whole (triangle of *Rules, Tools and Artefacts*, *Division of Labour*, and *Community*). We identified three papers which applied theory to patient involvement within the HEI system. Read et al. (2020) used PAR to demonstrate the benefits and challenges of extending partnership working beyond educational activities to the development of the processes needed to support involvement. Anderson et al. (2019) used Activity Theory (Engeström, 2001), and Cooper and Spencer-Dawe (2006) used Complexity Theory (Tosey, 2002) to highlight the complexity of patients leading teaching in IPE. These theoretically underpinned studies add a richness to our understanding of what it means to bring patients into a HEI system, both for faculty and for acceptance by students.

Although only three studies have begun to focus theoretically on these multifaceted components of the Activity Theory triangle (Anderson et al., 2019; Cooper & Spencer-Dawe, 2006; Read et al., 2020), all of the included papers had considered some aspects of the system. Most studies reported preparation and/or support for patients, yet student support and faculty development received little attention. Included studies primarily focused on the patient-student relationship, but neglected patient-educator partnerships. Indeed, Happell et al. (2014) highlighted a need to investigate the patient-educator dynamic, as educators are the gatekeepers to involvement. Few studies reported patient payment; descriptions were often brief and ambiguous. As such, our understanding of the policies and processes needed to support involvement and ensure a meaningful division of labour remains limited. Furthermore, we still do not know what it means to form a community of educators and patients, where patients feel and become valued as equal faculty partners.

We do recognise that theory might not hold all of the answers when considering the triangle of Activity Theory. Indeed, there are papers which are pragmatic in orientation that explore these aspects of involvement, considering patient payment, support, and preparation (Bassett et al., 2006; Felton & Stickley, 2004; Soklaridis et al., 2020). However, involving, supporting, and facilitating a sense of belonging for patients within HEIs inherently entails the navigation of power relations and hierarchies—something that these more pragmatic papers do acknowledge (Bassett et al., 2006; Felton & Stickley, 2004; Soklaridis et al., 2020). Theories derived from the wider social sciences literature that explore power (e.g., Foucault, 1980), group membership, and belonging (e.g., Tajfel & Turner, 2004; Wenger, 1998), could therefore undoubtedly contribute to our understanding of the *Rules, Tools and Artefacts, Division of Labour,* and *Community* within an Activity System for patient involvement in HEIs. We did identify one study (Rees et al., 2007) which applied Situated Learning (Lave & Wenger, 1991) to consider patients as ‘legitimate peripheral participants’ within ‘educational communities of practice’ (Wenger, 1998). While this paper was excluded as it did not stand the test of our theoretical quality tool, we agree that this is both an interesting and relevant theoretical perspective on patient involvement within education communities, which warrants further investigation.

Although conducted rigorously, our review has limitations. In restricting our review to papers published in English, within a 20-year timeframe, we may have missed earlier relevant research. We were forced to make pragmatic judgements to manage the sheer volume of papers retrieved, owing to the growth of this field. Throughout we faced concerns about the ubiquitous nature of the term ‘patient’ and its alternatives, and the absence of a consensus nomenclature to describe ‘patient involvement’ (Gordon et al., 2020). However, we used broad search parameters inclusive of all levels and areas of professional education. Scoping confirmed the validity of the final searches in that key papers were retained. Justifying excluded papers was challenging owing to the lack of detailed description by authors. We encountered several papers which appeared to have theoretical underpinnings but failed to explicitly articulate them. This limits the value of the evidence-base for researchers and educators (Gordon et al., 2020). Finally, reaching a consensus on the meaning of an intangible construct, like theory, as a research team was challenging. We approached this through iterative discussions about exemplar pilot papers: a strategy informed by previous research (Hean et al., 2016, 2018).

### Implications for future research

By identifying and aligning high-quality, theoretically underpinned papers to the components of Activity Theory, this review provides a map to guide future research and theoretical development in this field. We challenge educators and scholars to account for patient involvement in every aspect of the Activity System for patient involvement in professional health and social care education.

A recent BEME review has begun to look at a theoretical approach to patient involvement in teaching and learning about patient-centredness using a realist synthesis (De Groot et al., 2020). This is helpful and implies there are theoretical stances for all stakeholders—patients, students, and teachers. We do, however, need to further understand what patient involvement means for our educational communities. This might be identified using a range of other theoretical lenses to fill the gaps in our understandings of the interactions in an Activity System within HEIs, which in turn might guide policy (Department of Health & Social Care, 2021; GMC 2009; 2018). Furthermore, we lack a strong stakeholder perspective on these issues, in particular, a strong patient voice. Despite best intentions, we are still taking a largely ‘top down’ approach to working with patients in education and have a long way to go to achieve a true redistribution of power in HEI, as envisaged in the early citizen participation movement (Arnstein, 1969). Future research should seek the perspectives of patients who, through their active involvement in health professions education, have gained relevant experience and insight into what it means to become and belong within a community of healthcare educators. Finally, we urge authors in this field to write with more clarity about the contribution of theory to our understanding of what it means to work with patients in ways that enhance learning and foster inclusivity.

## Conclusion

The acceleration of work on patient involvement in all aspects of professional education is encouraging but is lacking in high-quality theoretical foundations. This review has, for the first time, identified the areas of patient involvement in professional education that have received high-quality theoretical consideration and those that require greater theoretical understanding. Future research must clearly articulate, operationalise and test the theoretical underpinnings of teaching and learning involving patients. Although theory has begun to illuminate the complexity of patient involvement, the literature fails to provide a conceptual framework to guide partnership working which can be adapted across different HEIs. Without such a framework, we risk remaining static and perpetuating paternalistic power relations (Regan de Bere & Nunn, 2016). Future research should explore theories that can help us to understand what it means to work in partnership with patients within education communities. This is arguably our greatest challenge, not least because partnership working is what policy asks of us (Department of Health, 2021; GMC, 2009; 2018), but because this is the only way to truly gain from patients’ authenticity.

## Supplementary Information

Below is the link to the electronic supplementary material.

Supplementary file1 (DOCX 95 kb)
