# Supplementary material for: A theoretical systematic review of patient involvement in health and social care education
Source: Adv Health Sci Educ Theory Pract. 2022 Jul 16;28(1):279–304. doi: 10.1007/s10459-022-10137-3 (PMC9992014; doi:10.1007/s10459-022-10137-3)
Supplement: Supplementary file 1 — Supplementary file1 (DOCX 95 kb) [file 10459_2022_10137_MOESM1_ESM.docx]

**Appendices**

**Appendix 1: BeHEMoTh Search Strategy and MEDLINE search statement.**

BeHEMoTH Search Strategy

| **Patient Involvement in Education**  *Behaviour (Be)* | **Type of involvement (i.e. faculty work undertaken)**  *Behaviour (Be)* | **Target audience of PPI in education**  *Health Condition or Context (HE)* | **Model or theory**  *Model or Theory (MoTh)* |
| --- | --- | --- | --- |
| **Medical Subject Headings (MeSH)**  **(“exp” indicates exploded MeSH)** | | | |
| exp Stakeholder Participation/ *OR*  exp Patient Participation/ *OR*  exp Community Participation/ | exp Teaching/ *OR*  exp School Admission Criteria/ *OR*  exp Formative Feedback/ OR  exp Simulation Training/ *OR*  exp Curriculum/ *OR*  exp Program Evaluation/ *OR*  exp Advisory Committees/ | exp Students, Health Occupations/ *OR*  exp Education, professional/  *OR*  exp Medicine/ *OR*  exp Nursing/ *OR* exp Social Work/ *OR*  exp Midwifery/ *OR*  Language Therapy/*OR*  Speech Therapy/ *OR*  Allied Health Personnel/ *OR*  exp Occupational Therapy/ *OR*  exp Pharmacists/ *OR*  exp Dentists/ *OR*  exp Clinical Psychology/ | Models, educational/ OR  Models, psychological/  OR  Models, organisational/ |
| **Free Text Search Terms** | | | |
| “Patient Public Involvement”.mp *OR*  (Patient* involv*” or “Patient* collaborat*” or “Patient* Participat*” or “Patient* integrat*” or “Patient* engag*” or “Patient* partner*” or “Patient* co-produc*”).mp *OR*  (“Carer* involv*” or “Carer* collaborat*” or “Carer* participat*” or “carer* integrat*” or “Carer* engag*” or “Carer* partner*” or “Carer* Co-produc*”).mp *OR*  (“Client* involv*” or “Client* collaborat*” or “Client* participat*” or “Client* integrat*” or “Client* engag*” or “Client* partner*” or “Client* co-produc*”).mp *OR*  (“Service user* involv*” or “Service user* collaborat*” or “Service user* participat*” or “Service user* integrat*” or “Service user* engag*” or “Service user* partner*” or “Service user* co-produc*”).mp *OR*  (“Consumer* involv*” or “consumer* collaborat*” or “Consumer* participat*” or “Consumer* integrat*” or “Consumer* engag*” or “Consumer* partner*” or “Consumer*co-produc*”).mp  *OR*  ((Lay or Disab*) adj3 (involv* or collaborat* or participat* or integrat* or co-produc* or engag* or partner*)).mp  *OR*  (“Patient teacher*” or “Patient educator*” or “patient led” or “Patient instructor*” or “Patient mentor*”).mp  *OR*  (Patient* adj3 Carer*).mp  *OR*  “Patient* and Carer* Group*”.mp | (teaching or learn* or training or assess* or recruit* or “student selection*” or “story-tell*” or feedback or simulat*) *OR*  ((course* or module* or curricul* or program*) AND (deliver* or design* or develop* or evaluat*)) | (Medic* or doctor* or nurs* or “social work*” or “social care” or midwif* or “speech and language therap*” or pharmac* or dentist* or “allied health” or physiotherapy* or “mental health nurs*” or “occupational therap*” or “health professional*” or “healthcare professional*” or interprofessional* or “multi professional*” or “clinical psychology*”).mp  *AND*  (educat* or undergraduate* or postgraduate* or student* or learner* or trainee* or “graduate entry” or graduate* or bachelors or “pre-registration” or competenc* or residenc* or “continuing professional development” or CPD or placement* or “post-qualif*”).mp | Theor*.mp *OR*  Model*.mp *OR*  Concept*.mp *OR*  Framework*.mp *OR*  Epistemolog*.mp *OR*  Ontolog*.mp *OR*  Underpin*.mp *OR*  Pedagog*.mp |

Search Statement from MEDLINE (05/01/21):

1. exp Stakeholder Participation/
2. exp Patient Participation/
3. “Patient public involvement”
4. (Patient* adj3 (involv* or collaborat* or participat* or integrat* or co-produc* or engag* or partner*)).mp
5. (Carer* adj3 (involv* or collaborat* or participat* or integrat* or co-produc* or engag* or partner*)).mp
6. (Client* adj3 (involv* or collaborat* or participat* or integrat* or co-produc* or engag* or partner*)).mp
7. (“Service user*” adj3 (involv* or collaborat* or participat* or integrat* or co-produc* or engag* or partner*)).mp
8. (Consumer* adj3 (involv* or collaborat* or participat* or integrat* or co-produc* or engag* or partner*)).mp
9. (Lay adj3 (involv* or collaborat* or participat* or integrat* or co-produc* or engag* or partner*)).mp
10. (Disabl* adj3 (involv* or collaborat* or participat* or integrat* or co-produc* or engag* or partner*)).mp
11. (“Patient teacher*” or “Patient educator*” or “Patient led” or “Patient instructor*” or “Patient mentor*”).mp
12. (Patient* adj3 Carer*).mp
13. “Patient* and carer* group*”.mp
14. **OR/ 1-13**
15. exp Teaching/
16. exp School Admission Criteria/
17. exp Formative Feedback/
18. exp Simulation Training/
19. exp Curriculum/
20. exp Program Evaluation/
21. (Teach* or learn* or train* or assess* or recruit* or “student selection” or “story tell*” or feedback or simulat*).mp
22. ((Course* or module* or curricul* or program*) AND (deliver* or design* or develop* or evaluat*)).mp
23. exp Advisory Committees/
24. (Committee* or governance).mp
25. **OR/15-24**
26. exp Students, Health Occupations/
27. exp Education, Professional/
28. **OR/ 26-27**
29. exp Medicine/
30. exp Nursing/
31. exp Social Work/
32. exp Midwifery/
33. Language Therapy/
34. Speech Therapy/
35. Allied Health Personnel/
36. exp Occupational Therapy/
37. exp Pharmacists/
38. exp Dentists/
39. exp Clinical Psychology/
40. (Medic* or doctor* or nurs* or “social work*” or “social care” or midwif* or “speech and language therap*” or pharmac* or dentist* or “allied health” or physiotherap* “mental health nurs*” or “occupational therap*” or “health professional*” or “healthcare professional*” or interprofessional* or “multi professional*” or “clinical psycholog*”).mp
41. OR/ 29-40
42. (educat* or undergraduate* or postgraduate* or student* or learner* or trainee* or “graduate entry” or graduate* or bachelors or “pre-registration” or competenc* or residenc* or “continuing professional development” or CPD or placement* or “post qualif*”).mp
43. AND/ 41 and 42
44. **OR/ 28 and 43**
45. Models, educational/ or Models, organizational/ or Models, psychological/
46. (theor* or model* or concept* or framework* or epistemolog* or ontolog* or underpin* or pedagog*).mp
47. **OR/ 45-46**
48. **AND/ 14 and 25 and 44 and 47**
49. **LIMIT 48 to English Language, 2000-2020, Journal Articles and clinical conferences.**

| **All Papers:** | **Y=1  N=0** | **Justification of Decision** (If “yes”, How? If “no”, Why?) |
| --- | --- | --- |
| **Paper citation** |  |  |
| **Theory or theories applied** |  |  |
| 1. ***Is there pragmatic adequacy?*** | | |
| i. There are clear concrete, feasible suggestions for how the theory proposed can actually be used in designing, delivering, or receiving curricula where patients and carers are involved **OR** how the proposed theory can be used to inform ways of working with patients and carers in educational settings (PA). |  |  |
| ii. The concepts proposed possess relevance to curriculum on paper/curriculum in action/curriculum received by the learner (PA) |  |  |
| iii. Will someone find this useful? Who will find this useful? (PA) |  |  |
| ***If answer “yes” to question 1 then proceed to question 2. If answer “no” to question 1 then consider exclusion from review*** | | |
| **All papers that answer “yes” to question 1:** | **Y=1 N=0** | If “yes”, How? If “no”, Why? |
| 1. ***Is theory clearly articulated?*** | | |
| i. Can you easily understand the abstract concepts presented and how they relate to practice? (P, T, PA.) |  |  |
| ii. Can you understand how the components of the theory relate to one another? (T, P) |  |  |
| iii. Are testable/useable propositions derived from the theory clearly presented? (T) |  |  |
| **Empirical papers only:** |  |  |
| 1. ***Is there evidence of operational and empirical adequacy?*** | | |
| i. Are theoretical claims tested or used empirically? (EA; T, OA) |  |  |
| ii. Are the methods of data collection appropriate to test/in the use of the theory or the propositions derived from the theory? (OA) |  |  |
| iii. Does the empirical evidence presented confirm the theory or propositions? Is there congruence between the theory and the evidence collected? (EA) |  |  |

**Appendix 2: Theoretical Quality Tool (Adapted from Hean et al, 2016)**

**Appendix 3: Excluded papers applying learning theories.**

| **Learning theory** | **Excluded Papers** | **Profession(s)** |
| --- | --- | --- |
| Adult Learning Theory | Hughes (2017) | Social Work |
| Cognitive Dissonance | Koufidis et al. (2020) | Medicine |
| Emancipatory Learning | River et al. (2017) | Social Work |
| Experiential Learning/ Reflection in and on action | Anderson and Thorpe (2010); Cumberland et al. (2019); Gee et al (2009); Valeras et al. (2013); Hayward and Li (2017); Kroll et al. (2008); Kubin and Wilson (2017); Ottewill et al. (2006); Read and Palmer (2013); Singleton (2006); Stojan et al. (2019) | Interprofessional Education; Physiotherapy; Nursing; Social Work; Medicine |
| Novice to Expert Theory | Kubin and Wilson (2017) | Nursing |
| Pedagogy of Discomfort | Coulter et al. (2013) | Social Work |
| Relational Learning | Langer et al. (2016) | Medicine |
| Schulman’s Conceptual Framework of Professional Development | Dyrbye et al (2007) | Medicine |
| Self-Efficacy | Agnew and Duffy (2010) | Social Work |
| Situated Learning/Communities of Practice | Hantman et al. (2013); Rees et al (2007); Towle and Godolphin (2013) | Social Work; Interprofessional Education; Medicine |
| Socio-Cultural Learning/ Social Cognitive Theory | Fong et al. (2019); Manninen et al. (2014) | Medicine; Nursing |
| Transformative Learning | Chua et al. (2020); Hughes (2017); Kittisarapong et al. (2016); Langlois et al. (2016); Rush (2008); Stacey et al. (2015) | Mental Health Nursing; Nursing; Interprofessional Education; Medicine; Social Work |

**References (Appendices)**

| Agnew, A., & Duffy, J. (2010). | Innovative approaches to involving service users in palliative care social work education. *Social Work Education*, *29*(7), 744-759. DOI:10.1080/02615471003657976 |
| --- | --- |
| Anderson, E. S., & Thorpe, L. (2010). | Learning together in practice: An interprofessional education programme to appreciate teamwork. *The Clinical Teacher*, *7*(1), 19-25. DOI:10.1111/j.1743-498X.2009.00331.x |
| Chua, I. S., Bogetz, A. L., Long, M., Kind, T., Ottolini, M., Lineberry, M., & Bhansali, P. (2021). | Medical student perspectives on conducting patient experience debrief interviews with hospitalized children and their families. *Medical Teacher*, *43*(4), 421-427. DOI:10.1080/0142159X.2020.1854707 |
| Coulter, S., Campbell, J., Duffy, J., & Reilly, I. (2013) | Enabling social work students to deal with the consequences of political conflict: Engaging with victim/survivor service users and a ‘pedagogy of discomfort’. *Social Work Education*, *32*(4), 439-452. DOI:10.1080/02615479.2012.668180 |
| Cumberland, D. M., Sawning, S., Church-Nally, M., Shaw, M. A., Branch, E., & LaFaver, K. (2019). | Experiential learning: Transforming theory into practice through the Parkinson’s disease buddy program. *Teaching and Learning in Medicine*, *31*(4), 453-465. DOI:10.1080/10401334.2019.1580583 |
| Dyrbye, L. N., Harris, I., & Rohren, C. H. (2007) | Early clinical experiences from students' perspectives: A qualitative study of narratives. *Academic Medicine*, *82*(10), 979-988. DOI:10.1097/ACM.ob013e318149e29c |
| Fong, S., Tan, A., Czupryn, J., & Oswald, A. (2019) | Patient-centred education: How do learners’ perceptions change as they experience clinical training?. *Advances in Health Sciences Education*, *24*(1), 15-32. DOI:10.1007/s10459-018-9845-y |
| Gee, M., Ager, W., & Haddow, A. (2009) | The caring experience: Learning about community care through spending 24 hours with people who use services and family carers. *Social Work Education*, *28*(7), 691-706. DOI:10.1080/02615470802404200 |
| Hantman, S., Oz, M. B., Gutman, C., & Criden, W. (2013). | Bringing older adults into the classroom: The sharing community model. *Gerontology & Geriatrics Education*, *34*(2), 135-149. DOI:10.1080/02701960.2012.679372 |
| Hayward, L. M., & Li, L. (2017) | Sustaining and improving an international service-learning partnership: Evaluation of an evidence-based service delivery model. *Physiotherapy Theory and Practice*, *33*(6), 475-489. DOI:10.1080/09593985.2017.1318425 |
| Hughes, M. (2017) | What difference does it make? Findings of an impact study of service user and carer involvement on social work students’ subsequent practice. *Social Work Education*, *36*(2), 203-216. DOI:10.1080/02615479.2016.1274725 |
| Kittisarapong, T., Blatt, B., Lewis, K., Owens, J., & Greenberg, L. (2016) | Interdisciplinary workshop to increase collaboration between medical students and standardized patient instructors in teaching physical diagnosis to novices. *MedEdPORTAL*, *12*. DOI:10.15766/mep_2374-8265.10411 |
| Koufidis, C., Manninen, K., Nieminen, J., Wohlin, M., & Silén, C. (2020). | Grounding judgement in context: A conceptual learning model of clinical reasoning. *Medical Education*, *54*(11), 1019-1028. DOI:10.1111/medu.14222 |
| Kroll, T., Groah, S., Gilmore, B., & Neri, M. (2008) | Consumer-Directed Teaching of Health Care Professionals Involved in the Care of People With Spinal Cord Injury: The Consumer–Professional Partnership Program. *The Journal of Continuing Education in Nursing*, *39*(5), 228-234. DOI: 10.3928/00220124-20080501-05 |
| . Kubin, L., & Wilson, C. E. (2017). | Effects of community volunteer children on student pediatric assessment behaviors. *Clinical Simulation in Nursing*, *13*(7), 303-308. DOI:10.1016/j.ecns.2017.04.011 |
| Langer, T., Martinez, W., Browning, D. M., Varrin, P., Lee, B. S., & Bell, S. K. (2016). | Patients and families as teachers: A mixed methods assessment of a collaborative learning model for medical error disclosure and prevention. *BMJ Quality & Safety*, *25*(8), 615-625. DOI:10.1136/bmjqs-2015-004292 |
| Langlois, S., Teicher, J., Derochie, A., Jethava, V., Molley, S., & Nauth, S. (2017). | Understanding partnerships with patients/clients in a team context through verbatim theater. *MedEdPORTAL*, *13*. DOI:10.15766/mep_2374-8265.10625 |
| Manninen, K., Henriksson, E. W., Scheja, M., & Silén, C. (2014) | Patients’ approaches to students’ learning at a clinical education ward-an ethnographic study. *BMC Medical Education*, *14*(1), 1-8. DOI:10.1186/1472-6920-14-131 |
| Ottewill, R., Demain, S., Ellis-Hill, C., Greenyer, C. H., & Kileff, J. (2006). | An expert patient-led approach to learning and teaching: The case of physiotherapy. *Medical Teacher*, *28*(4), 120-126. DOI:10.1080/0142590600726698 |
| Read, J., & Palmer, R. (2013) | A stroke staff training programme involving expert patients: A case study of its impact on staff and service development. *International Practice Development Journal*, *3*(2). DOI:10.19043/idpj.32.003 |
| Rees, C. E., Knight, L. V., & Wilkinson, C. E. (2007). | “User involvement is a sine qua non, almost, in medical education”: Learning with rather than just about health and social care service users. *Advances in Health Sciences Education*, *12*(3), 359-390. DOI:10.1007/s10459-006-9007-5 |
| River, D. H., Thakoordin, J. M., & Billing, L. (2017) | Creativity in social work education and practice: Reflections on a Survivor Arts Project. *Social Work Education*, *36*(7), 758-774. DOI:10.1080/02615479.2016.1266320 |
| Rush, B. (2008) | Mental health service user involvement in nurse education: A catalyst for transformative learning. *Journal of Mental Health*, *17*(5), 531-542. DOI:10.1080/09638230802053383 |
| Singleton, J. L. (2006) | Infusing gerontology throughout the BSW curriculum. *Journal of Gerontological Social Work*, *48*(1-2), 31-46. DOI:10.1300/J083v48n01_03 |
| Stacey, G., Oxley, R., & Aubeeluck, A. (2015) | Combining lived experience with the facilitation of enquiry‐based learning: a ‘trigger’ for transformative learning. *Journal of Psychiatric and Mental Health Nursing*, *22*(7), 522-528. DOI:10.1111/jpm.12228 |
| Stojan, J. N., Sun, E. Y., & Kumagai, A. K. (2019). | Persistent influence of a narrative educational program on physician attitudes regarding patient care. *Medical Teacher*, *41*(1), 53-60. DOI:10.1080/014259X.2018/1436755 |
| Towle, A., & Godolphin, W. (2013). | Patients as educators: Interprofessional learning for patient-centred care. *Medical Teacher*, *35*(3), 219-225. DOI:10.3109/0142159X.2012.737966 |
| Valeras, A., Gunn, W., & Valeras, A. (2013). | An innovative model for systems-based curriculum: The complex continuity clinic. *The International Journal of Psychiatry in Medicine*, *45*(4), 377-387. DOI:10.2190/PM.45.4.h |
